# Extracellular vesicle-associated miRNAs are an adaptive response to gestational diabetes mellitus

**DOI:** 10.1186/s12967-021-02999-9

**Published:** 2021-08-20

**Authors:** Soumyalekshmi Nair, Dominic Guanzon, Nanthini Jayabalan, Andrew Lai, Katherin Scholz-Romero, Priyakshi Kalita de Croft, Valeska Ormazabal, Carlos Palma, Emilio Diaz, Elizabeth A. McCarthy, Alexis Shub, Jezid Miranda, Eduard Gratacós, Fátima Crispi, Gregory Duncombe, Martha Lappas, H. David McIntyre, Gregory Rice, Carlos Salomon

**Affiliations:** 1 Exosome Biology Laboratory, Centre for Clinical Diagnostics, UQ Centre for Clinical Research, Royal Brisbane and Women’s Hospital, Faculty of Medicine, The University of Queensland, Building 71/918, Herston, QLD 4029 Australia; 2 Faculty of Biological Sciences, Pharmacology Department, University of Concepcion, Concepción, Chile; 3 Faculty of Medicine, Department of Obstetrics and Gynaecology, University of Concepcion, Concepción, Chile; 4 Department of Obstetrics and Gynaecology, University of Melbourne, Melbourne, Australia; 5 Mercy Hospital for Women, 163 Studley Road, Heidelberg, VIC 3084 Australia; 6 Fetal Medicine Research Center, BCNatal-Barcelona Center for Maternal-Fetal and Neonatal Medicine (Hospital Clínic and Hospital Sant Joan de Déu), Institut Clínic de Ginecologia Obstetricia i Neonatologia, Universitat de Barcelona, Centre for Biomedical Research on Rare Diseases (CIBER-ER), Barcelona, Spain; 7 Mater Research, Faculty of Medicine, University of Queensland, Mater Health, South Brisbane, Australia

**Keywords:** Pregnancy, Exosomes, miRNAs, Insulin resistance, Skeletal muscle

## Abstract

**Background:**

Gestational diabetes mellitus (GDM) is a serious public health issue affecting 9–15% of all pregnancies worldwide. Recently, it has been suggested that extracellular vesicles (EVs) play a role throughout gestation, including mediating a placental response to hyperglycaemia. Here, we investigated the EV-associated miRNA profile across gestation in GDM, assessed their utility in developing accurate, multivariate classification models, and determined the signaling pathways in skeletal muscle proteome associated with the changes in the EV miRNA profile.

**Methods:**

Discovery: A retrospective, case–control study design was used to identify EV-associated miRNAs that vary across pregnancy and clinical status (*i.e.* GDM or Normal Glucose Tolerance, NGT). EVs were isolated from maternal plasma obtained at early, mid and late gestation (n = 29) and small RNA sequencing was performed. Validation: A longitudinal study design was used to quantify expression of selected miRNAs. EV miRNAs were quantified by real-time PCR (cases = 8, control = 14, samples at three times during pregnancy) and their individual and combined classification efficiencies were evaluated. Quantitative, data-independent acquisition mass spectrometry was use to establish the protein profile in skeletal muscle biopsies from normal and GDM.

**Results:**

A total of 2822 miRNAs were analyzed using a small RNA library, and a total of 563 miRNAs that significantly changed (p < 0.05) across gestation and 101 miRNAs were significantly changed between NGT and GDM. Analysis of the miRNA changes in NGT and GDM separately identified a total of 256 (NGT-group), and 302 (GDM-group) miRNAs that change across gestation. A multivariate classification model was developed, based on the quantitative expression of EV-associated miRNAs, and the accuracy to correctly assign samples was > 90%. We identified a set of proteins in skeletal muscle biopsies from women with GDM associated with *JAK-STAT* signaling which could be targeted by the miRNA-92a-3p within circulating EVs. Interestingly, overexpression of miRNA-92a-3p in primary skeletal muscle cells increase insulin-stimulated glucose uptake.

**Conclusions:**

During early pregnancy, differently-expressed, EV-associated miRNAs may be of clinical utility in identifying presymptomatic women who will subsequently develop GDM later in gestation. We suggest that miRNA-92a-3p within EVs might be a protected mechanism to increase skeletal muscle insulin sensitivity in GDM.

**Supplementary Information:**

The online version contains supplementary material available at 10.1186/s12967-021-02999-9.

## Background

Gestational Diabetes Mellitus (GDM) is defined as glucose intolerance with onset or first recognition during pregnancy [1]. With a worldwide prevalence of 9–15%, GDM has been recognized as the most common medical complication during pregnancy [2]. The hyperglycemic intrauterine environment in GDM pregnancies increases the risk of excessive fetal growth, shoulder dystocia, caesarean section and neonatal hypoglycemia [3]. Furthermore, women with a history of GDM have increased risk of developing type 2 diabetes and cardiovascular disease, whilst their offspring have increased risks for early onset type 2 diabetes and obesity [4]. The pathophysiology of GDM, however, is not well understood. Over the last decade, our studies involving extracellular vesicles (EVs) have shown that they may have a key role in GDM [5–7].

EVs are membrane-enclosed vesicles secreted from a wide range of cells, including cells within the human placenta. Interestingly, the various subpopulations of EVs share similar physical and biochemical characteristics, which makes it difficult to separate pure populations of EVs, bringing about considerable heterogeneity in the EV field [8]. Currently, there is a lack of consensus regarding the nomenclature to describe different subtypes of EV. Importantly, efforts are in place to classify these vesicles precisely, based on their morphology, surface markers, biogenesis and enrichment/purification methods [9, 10]. The International Society of Extracellular Vesicles (ISEV) endorses the term “extracellular vesicle” for particles enclosed by a lipid bilayer, and devoid of a nucleus, and recommends using the term “small EV” for EV < 100 to 200 nm in diameter (commonly known as exosomes), and “medium/large EV” for those > 200 nm [10]. The EV field has been focused on the ability of these vesicles to incorporate specific bioactive molecules (*e.g*. proteins and miRNAs) into their cargo and deliver them to target cells, leading to changes in recipient cell functions [11, 12]. Hence, EVs act as critical mediators of cell-to-cell communication and important modulators of physiological and pathological conditions.

The concentration of circulating small EVs (sEVs) increases progressively across normal pregnancy from first to third trimester [13]. Notably, it has been demonstrated that sEVs isolated from pregnant women can induce the secretion of cytokines from endothelial cells and that this effect is greater when sEVs were isolated from GDM pregnancies [5]. Recently, we reported that hyperglycemia increases the release of sEVs from first trimester primary human trophoblast (PHT) cells [6]. The presence and intercellular transfer of signaling molecules such as miRNAs via sEVs makes them attractive biomarkers for disease onset and monitoring.

miRNAs are a novel class of small non-coding RNAs, approximately 21 nucleotides long and are key mediators of mRNA translational regulation in cells [14]. miRNAs play a pivotal role in the development and maintenance of pregnancy by mediating diverse aspects such as placental development, angiogenesis, immune tolerance and feto-maternal communication [15]. Several studies have identified maternal circulating- and tissue-specific miRNA signatures in GDM at different stages of gestation and identified their potential effect in mediating the pathophysiology of glucose intolerance [16, 17].

Pregnancy is characterized by changes in the maternal metabolism which involves changes in insulin sensitivity, insulin secretion and lipolysis. The insulin-stimulated glucose disposal declines by 40–60% in late gestation compared to pre-pregnancy [18]. Skeletal muscle is the principal site for glucose uptake in the body and GDM leads to changes in the insulin signalling and associated molecules in skeletal muscle [19]. This is influenced by placental hormones and cytokines such as placental growth hormone and TNFα [19]. Previously, we have reported that the chorionic villi-derived sEVs can alter the glucose uptake in skeletal muscles [7]. The relationship between circulating sEVs miRNA profile and changes in skeletal muscle reflecting maternal metabolic adaptation in GDM is yet be characterized.

To date, there is a paucity of data defining changes in the miRNA content of EV as a function of gestational age and GDM. Thus, the hypotheses to be tested in this study are (i) the miRNA content of maternal plasma sEVs is differentially expressed across gestation, and in association with GDM; (ii) the quantification of sEV-associated miRNA expression in early pregnancy plasma is of utility in developing accurate, multivariate classification models, and (iii) to characterize the changes in proteomic profile in the skeletal muscles in GDM using a quantitative mass spectrometric (MS) approach and identify the proteins and pathways targeted by circulating sEV-associated miRNAs using bioinformatic analysis.

## Materials and methods

### Study design

Discovery cohort: A retrospective, cross-sectional case–control study design was used to identify EV-associated miRNAs that vary with gestational age and clinical status (i.e. GDM or NGT). Validation cohort: To further characterize gestational age and GDM-associated variation in sEV miRNA, a retrospective, longitudinal, case–control study design was used to quantify expression of a suite of miRNAs.

### Ethics and data quality assurance

All experimental procedures were conducted within an ISO17025 accredited (National Association of Testing Authorities, Australia) research facility. All data were recorded within a 21 Code of Federal Regulation (CFR) part 11 compliant electronic laboratory notebook (Lab Archives, Carlsbad, CA 92008, USA). The project was approved by the Human Research Ethics Committees of the Royal Brisbane and Women’s Hospital, and the University of Queensland (HREC/11/QRBW/342), the Mercy Hospital for Women (HREC R10/16 and R04/29), Ethics Committees of the health service Concepcion and Universidad de Concepcion (Chile, ORD002373), and from the Institutional Research and Ethics Committee of the University of Barcelona, and BCNatal approved the study protocol (review board 2014/7154). Written informed consent was obtained from all women participating in the study.

### Study group and samples

#### Discovery cohort

Samples were collected in Australia at the Royal Brisbane and Women’s Hospital, and the Mercy Hospital for Women. EVs were isolated from maternal plasma obtained during early (< 18 weeks, cases = 2; controls = 2), mid (22–28 weeks, cases = 9; controls = 8) and late (37–40 weeks, cases = 4; controls = 4) gestation and small RNA sequencing was used to identify EV-associated miRNAs transcripts (Additional file 1: Fig. S1). GDM was diagnosed by testing with a three sample 75 g OGTT at 24–28 weeks, with cut-offs set according to ADIPS and WHO recommendations (GDM diagnosis – at least one elevated venous plasma glucose reading >  = 5.1 mmol/L fasting, >  = 10.0 mmol/L at 1 h, >  = 8.5 mmol/L at 2 h) [20, 21]. All samples were stored at − 80 °C prior to miRNA sequencing.

#### Validation cohort

Samples were collected at Hospital Clínic and Hospital Sant Joan de Deu, Barcelona, Spain. sEV miRNAs were quantified by real time PCR (cases = 8, control = 14 matched for gestational age, parity, and BMI), serially sampled at early, mid, and late gestation) (Additional file 1: Fig. S2). Two step GDM screening was performed between 24 and 28 weeks of gestation using a 50 g, 1-h glucose load test. Venous plasma glucose (VPG) ≥ 140 mg/dL were considered screen positive. GDM was diagnosed using a 100 g, 3-h oral glucose tolerance test (OGTT) using the criteria proposed by the National Diabetes Data Group (NDDG), namely fasting, 1-h, 2-h, and 3-h plasma glucose levels ≥ 105, 190, 165, and 145 mg/dL, respectively, with two elevated values required for GDM diagnosis. The individual and combined classification efficiencies of miRNAs were summarized by Receiver Operating Characteristic (ROC) curve and multivariate linear regression analysis. Samples from patients with normal pregnancy, or GDM without other metabolic and obstetric complications, were included in this study.

### Isolation and characterization of EV

EVs were isolated from plasma (1 mL) as previously described [5]. In brief, plasma was diluted with an equal volume of PBS (pH 7.4) and centrifuged at 2000×*g* for 30 min at 4 °C (Sorvall®, high speed microcentrifuge, fixed rotor angle: 90°, Thermo Fisher Scientific Ins., Asheville, NC, USA,). The 2000×*g* supernatant fluid was then centrifuged at 12,000×*g* for 45 min at 4 °C (Sorvall, high speed microcentrifuge, fixed rotor angle: 90°). The resultant supernatant fluid (2 mL) was transferred to an ultracentrifuge tube (Beckman, 10 ml) and centrifuged at 100,000×*g* for 2 h (Sorvall, T-8100, fixed angle ultracentrifuge rotor). The pellet was resuspended in PBS (10 mL) and filtered through a 0.22 μm filter (Steritop™, Millipore, Billerica, MA, USA) and then centrifuged at 100,000×*g* for 2 h. The 100,000 g pellet was resuspended in 500 μL of PBS and layered on top of a discontinuous iodixanol gradient containing 40% (w/v), 20% (w/v), 10% (w/v) and 5% (w/v) iodixanol (solutions were made by diluting a stock solution of OptiPrep™ (60% (w/v) aqueous iodixanol from Sigma-Aldrich) and centrifuged at 100,000×*g* for 20 h. Fractions were collected manually from top to bottom (with increasing density), diluted with PBS and centrifuged at 100,000×*g* for 2 h at 4 °C. Finally, the pellet containing the enriched sEVs population was resuspended in 50 μL PBS. The density of each fraction was measured in a control OptiPrep™ gradient tube by determining the absorbance at 244 nm. sEV-containing fractions (density 1.12 to 1.19 g/mL) were combined in a single tube and further characterized by size distribution, abundance of proteins associated with exosomes (*i.e.* CD63, sc15363; Flotilin-1, sc25506; and TSG101, EPR7130), and a negative control for Grp94 (20292T)), and morphology according to the recommendations of the International Society of Extracellular Vesicles [22], using Nanoparticle Tracking Analysis (NTA), Western blot analysis, and electron microscopy, respectively.

### sEV RNA isolation and next generation sequencing

sEV RNA was extracted using the RNeasy Mini Kit 50 (Qiagen, Australia) and TRIzol LS Reagent (Life Technologies, Australia). RNaseA treatment (100 ng/mL, Qiagen, Australia, 37 °C for 10 min) was used to select only RNA encapsulated within sEVs as previously described [23]. The total RNA yield (comprising of mostly small RNA), composition and quality was analyzed using the Agilent 2,100 Bioanalyser for small RNA profiles. Sequencing libraries were generated using the TruSeq® SmallRNA Library Prep Kit, according to the manufacturer’s instructions and as we previously described [24]. The elution containing the pooled DNA library was further processed for cluster generation and sequencing using NextSeq 500 High Output kit 75 cycles and Illumina NextSeq 500 sequencing platform, respectively. Sequencing data have been deposited in the Gene Expression Omnibus (GEO) database with accession number GSE114860. The resulting miRNA counts were filtered, and only miRNAs with at least 1 count in every sample were retained. The DESeq2 package (version 1.18.1) in R (version 3.2.2) was used to normalize the raw miRNA counts by applying the median ratio method.

### Gene target and gene ontology analysis

Gene targets regulated by statistically significant miRNAs were identified using the CyTargetLinker application (version 3.0.1), and gene ontology analysis was performed using the BiNGO (version 3.0.2) application in Cytoscape as previously described [24]. To visualise the protein–protein interaction networks, the Search Tool for the Retrieval of Interacting Genes/Proteins (STRING; string-db.org/) was used with a minimum interaction score of 0.4 [25].

### Real-time PCR

Reverse transcription was performed on 156 ng of total RNA using the miScript II RT Kit (QIAGEN, Valencia, CA, USA) using the HiSpec buffer. Real-time PCR was performed with the miScript SYBR Green Kit (QIAGEN, Valencia, CA, USA). Forward primers (miScript primer assays, QIAGEN, Valencia, CA, USA) designed to detect the following mature miRNAs were used: hsa-let-7i-5p (MS00003157), hsa-miR-10a-5p (MS00031262),, hsa-miR-151b (MS00037513), hsa-miR-16–2-3p (MS00008813), hsa-miR-16-5p (MS00031493), hsa-miR-1910-5p (MS00016464), hsa-miR-423-5p (MS00009681), hsa-miR-92a-3p (MS00006594) and hsa-miR-92b-3p (MS00032144). The reactions were performed in triplicate using the QuantStudio 3 real-time PCR system (USA) with the following conditions: 95 °C for 15 min, 60 amplification cycles of 94 °C for 15 s, 55 °C for 30 s and 70 °C for 30 s. The miRNA expression was normalized using the ∆∆CT method with the recommended housekeeping gene RNU6B (MS00033740). RNU6B expression was consistent in all the samples both within and across experimental conditions. No statistically significant differences (p > 0.05) in the expression of RNU6B between sEVs and/or cells samples measured by Standard Deviation of CT were identified.

### Liquid chromatography–mass spectrometry (LC–MS/MS) in skeletal muscle tissues

Skeletal muscle (from the *rectus pyramidalis*) was obtained from NGT (n = 9) and GDM (n = 18) women who delivered a healthy, singleton infant at term (37–41 weeks of gestation) via elective caesarean in the absence of labor. The protein expression in skeletal muscle tissue in NGT and GDM was identified by LC–MS/MS. For this, the protein lysates and its tryptic peptide digests were subjected to OFFGEL fractionation according to their pI in a 3100 OFFGEL Fractionator (Agilent Technology) into 24 fractions. Protein quantification was performed by data-independent acquisition (Sequential Windowed Acquisition of All Theoretical Mass Spectra [SWATH]) as previously described [26].

### MiRNA transfection and PCR array for JAK-STAT signaling

Primary skeletal muscle cultures were developed from skeletal muscle tissues obtained from NGT women (n = 6) at term elective Caesarean section. Briefly, the skeletal muscle tissue was dissected from fat and connective tissue and minced into fine pieces. The tissue was digested using trypsin and single cell suspension obtained using a cell strainer. The cells were collected by centrifugation at 550*g* for 10 min and cultured in gelatin-coated flasks. Following this, the cells were differentiated for 5 days and miRNA transfection was performed using Lipofectamine 3000 (Thermo Fisher Scientific, Australia) as per the manufacturers protocol. Briefly, skeletal muscle myotubes were transfected with 10 nM miR-92a-3p mimic (MSY0000092, Qiagen, Australia) and All Stars Negative Control (Qiagen, Australia). After 72 h of transfection, RNA was extracted from cells using RNeasy Mini Kit 50 (Qiagen, Australia) and Qiazol Reagent (Life Technologies, Australia) as per manufacturer’s protocol. 500ngs of RNA was used for cDNA synthesis using RT^2^ First Strand kit (Qiagen, Australia). A human JAk/STAT signlaing pathway RT^2^ Profiler PCR array (PAHS-039YA-6, Qiagen Australia) was used to screen a panel of 84 genes representative of JAK/STAT pathway. The Qiagen RT2 Profiler PCR Arrays in 96-well plates contained primer assays for 84 pathway- or disease-focused genes and 5 housekeeping genes. In addition, there were controls for genomic DNA, reverse-transcription and positive PCR controls. The house keeping genes were beta actin, beta-2-microglobulin, glyceraldehyde-3-phosphate dehydrogenase (GAPDH), Hypoxanthine phosphoribosyltransferase 1 (HGPRT/HPRT) and Ribosomal protein, large, P0 (L10E/LP0/P0/PRLP0/RPP0). cDNA was added to RT^2^ SYBR Green/ROX PCR Master Mix (Qiagen, Australia) and subsequently added to the PCR arrays (RT^2^ Profiler PCR array, Qiagen, Australia). qPCR was performed on Applied Biosystems QuantStudio 3 (Applied Biosystems, Thermo Fischer Sceintific, Inc.) and the real-time amplification data (Ct values) were obtained. Analysis was performed using the GeneGlobe Analysis Centre (Qiagen, Australia). Fold-Change (2^(- ΔΔCT)) was calculated by dividing the normalized gene expression (2^(- ΔCT)) in the test sample by the normalized gene expression (2^(- ΔCT)) in the control sample.

### SOCS5 3’ Untranslated Region (UTR) Luciferase Assay

To determine the transfection efficiency of miR-92a-3p on primary skeletal muscle cells, the untranslated region of human SOCS5 was cloned into a firefly/renilla Duo-Luciferase reporter vector (pEZX-MT06) (GeneCopoeia, Rockville, MD, USA). Primary human skeletal muscle cells (Lonza Australia Pty Ltd, Toowong, QLD) were differentiated for five days and transfected using Lipofectamine3000 (Thermo Fisher Scientific, Australia). Transfections were performed using 150 ng of dual luciferase reporter plasmids and a 10 nM concentration of synthetic mir-92a-3p mimic (MSY0000092, Qiagen, Australia). Transfections with pEZX-MT06 control plasmid (GeneCopoeia, Rockville, MD, USA) and All Stars Negative Control miRNA (Qiagen, Australia) were used as controls. Dual luciferase assays were performed Luc-Pair Duo-Luciferase Assay Kit 2.0 (GeneCopoeia, Rockville, MD, USA) as per the manufacturer’s protocol at 48 and 72 h. Firefly luciferase was normalized to Renilla luciferase control.

### MiRNA transfection and insulin-stimulated glucose uptake assay

Primary human skeletal muscle cells (Lonza Australia Pty Ltd, Toowong, QLD) were cultured and differentiated for 3 days and transfected with 10 nM synthetic miR-92a-3p mimic (MSY0000092, Qiagen, Australia) using Lipofectamine 3000 (Thermo Fisher Scientific, Australia). At 72 h, cells were washed with PBS and the media was replaced with a serum-free, glucose-free DMEM (Invitrogen, Australia) for 2 h. Cells were stimulated with 1uM insulin for 1 h and susequently treated with 0.1 mM 2DG for 30 min. Cells were lysed and 2-Deoxyglucose-6-phophate (2-DGP) was measured using the Glucose Uptake-Glo Assay kit (Promega) as per the manufacturers protocol.

### Statistical analysis

Variation in miRNA expression data were analyzed by ANOVA with variance partitioned between trimester and clinical status. Two statistical approaches to analyse the miRNA sequencing data were used. First, data was analyzed by two-way ANOVA (with LSD post-hoc testing to discriminate among the means) to determine the interaction between gestational age and GDM. Second, data was also categorized into NGT and GDM, and a one-way ANOVA with post-hoc test on each was analyzed to determine the overlap. Non-normally distributed data were logarithmically transformed before analysis. All analyses were performed using the R statistical software. Statistical significance was denoted by False Discovery Rate (FDR)-adjusted p < 0.05.

Quantitative miRNA expression data was analyzed using commercially available software (STATA ver 15.1, STATA Corp. College Station, TX, USA). Shaprio-Wilk [27] tests were used to assess the normality of data distributions. Non-parametric statistical tests were used where data distributions significantly deviated from normality. Between group differences were assessed by Two-sample Wilcoxon Rank-Sum tests. [28] Variation in miRNA expression within case and control cohorts was assessed using a Panel Data Analysis and Random Effects Generalized Least Squares (RE-GLS) models. [29] Statistical significance was ascribed when p < 0.05.

#### Modeling analysis

Linear mixed modeling was performed using the lme4 package implemented in R. Statistical analysis (likelihood ratio test) was performed comparing the full model, that included gestational age, BMI and OGTT variables, compared to a simpler model, that excluded these variables. This was performed to understand whether gestational age, BMI or glucose had effects on miRNA expression. P-value < 0.05 was chosen as the cutoff for statistical significance.

#### Power calculation

With respect to the expression in sEVs, miRNAs (using the mean and standard deviation) for the discovery phase with the experimental designs described above, at a significance level of α = 0.05, with size effect of 1 and NGT to GDM sample ratio of 1:1 achieves a power of 0.75. With respect to sEVs miRNAs for the validation phase, using the experimental designs described above, at a significance level of α = 0.01, with size effect of 2.0 and NGT to GDM sample ratio of 2:1 achieves a power of 0.80 (Additional file 1: Table S1).

## Results

### Isolation and characterization of EVs

**Fig. 1:**
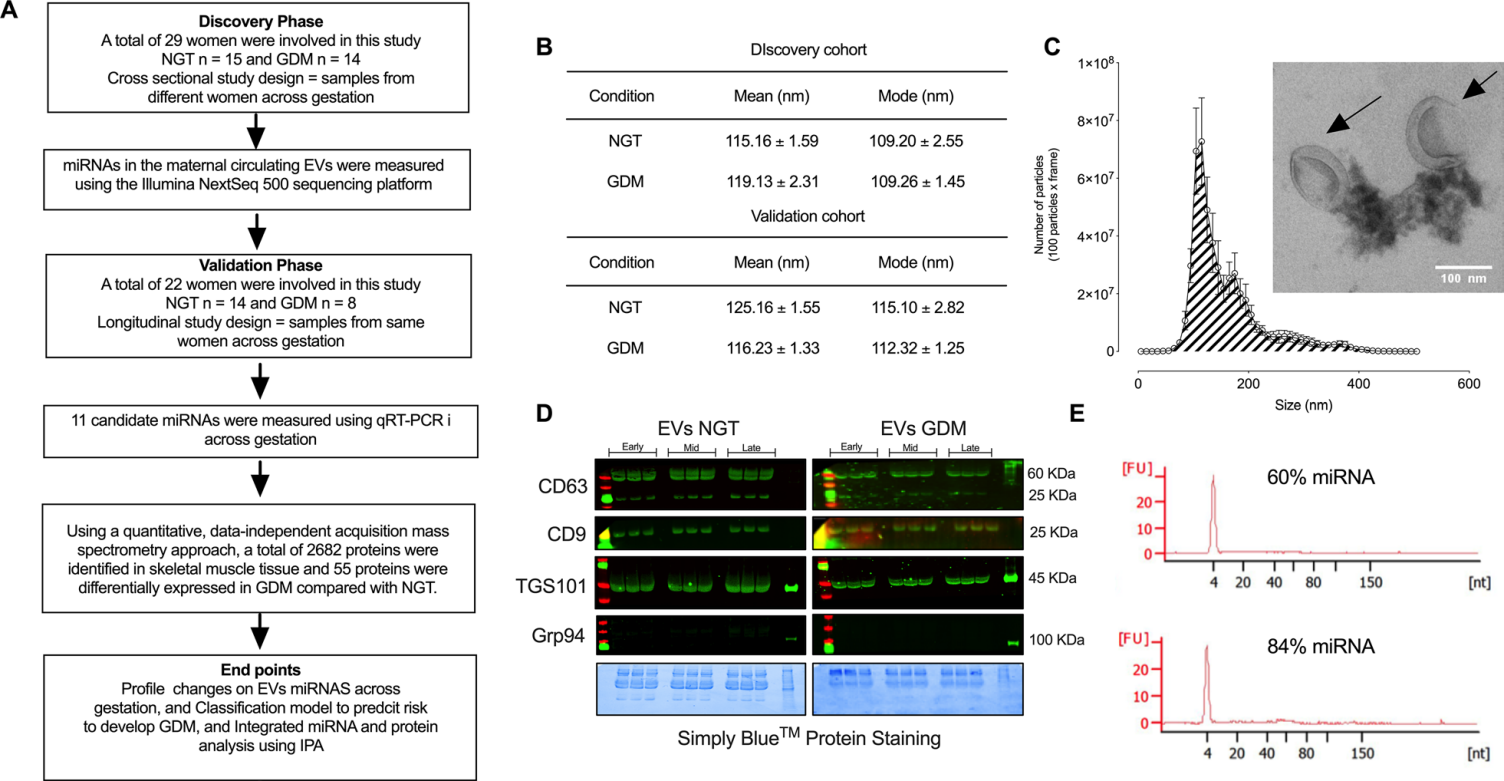
Isolation and characterization of EVs. **A** Outline of the study design. EVs were isolated from plasma obtained from NGT and GDM pregnancies across gestation. **B** Mean and mode of the vesicles isolated from NGT and GDM patients in the discovery and validation cohort. **C** Graphical representation of the vesicle size distribution using a NanoSight NS500 instrument, all gestational age combined in NGT and GDM, and representative image of electron micrograph of exosomes. **D** Representative Western blot for enriched EVs markers, CD63, CD9 and TSG101, and negative control Grp94, for EVs isolated from normal and GDM at different time points during pregnancy. **E** Representative images of small RNA profile extracted from EVs from NGT (up image), and GDM (down image). In C insert, Scale bar 100 nm. In **A**–**C**, **E**, none of the experiments performed were significantly different between NGT vs. GDM

Demographic data of all participants involved in this study are summarized in Table 1 and Additional file 1: Table S2. The study design is presented in Fig. 1A. Particles with a diameter of ~ 100 nm were identified using Nanoparticle Tracking Analysis and displayed cup-shaped morphology on Transmission Electron Microscopy (Fig. 1B, C, and Additional file 1: Table S3). The vesicles obtained were positive for proteins associated with small EVs such as CD63, CD9 and TSG101 (Fig. 1D). The EV enriched fraction was negative for the endoplasmic reticulum marker Grp94 (Fig. 1D). No statistically-significant differences were observed in nanoparticle size distribution, protein abundance and morphology between NGT and GDM. A representative analysis of the percentage of miRNA in sEVs isolated from NGT and GDM is presented in Fig. 1E and varied between 60 ± 7.0% and 84.3 ± 12%.

**Table 1:** Clinical characteristics of patients and newborns in the validation cohort

	NGT (n = 14)	GDM (n = 8)	p values
Maternal baseline characteristics			
Age (years)	31.6 ± 5.24	36.9 ± 3.02	0.003
Race			0.256
Chinese	1 (5.56%)	0	
European	8 (44.4%)	8 (88.9%)	
Indian–Pakistan	3 (16.7%)	0	
Latin–American	5 (27.8%)	1 (11.1%)	
Philipina	1 (5.56&)	0	
Height (cms)	162 ± 6.13	162 ± 3.52	0.929
Pre-gestational weight (kg)	66.6 ± 13.9	61.7 ± 13.7	0.398
Pre-gestational BMI	25.3 ± 4.67	23.5 ± 5.46	0.411
Screening and diagnostic results			
Gestational age O´Sullivan test	23.6 ± 3.6	24.4 ± 1.25	0.52
O’Sullivan test result (mmol/L)	7.0 ± 1.67	9.70 ± 1.88	0.004
Gestational age at OGTT	26.4 ± 0.55	26.0 ± 0.75	0.266
Fasting OGTT (mmol/L)	4.38 ± 0.25	4.58 ± 0.53	0.342
1 h OGTT (mmol/L)	8.22 ± 1.16	11.1 ± 1.15	< 0.001
2 h OGTT (mmol/L)	6.64 ± 2.14	10.0 ± 1.45	0.005
3 h OGTT (mmol/L)	5.21 ± 2.01	7.79 ± 1.50	0.016
Delivery data			
Gestational age at delivery (weeks)	39.6 ± 1.2	39.1 (± 1.22)	0.298
Fetal sex (male/female)	11/7	3/6	0.236
Birthweight (grams)	3480 ± 148	3154 ± 100	0.208
Birthweight percentile	47.8 ± 31.4	43.2 (± 25.1)	0.684
Route of delivery			0.363
Cesarean	6 (33.3%)	1 (11.1%)	
Vaginal	12 (66.7%)	8 (88.9%)	

Data are presented as mean ± SD. All pregnancies were normotensive, and without intrauterine infection or any other medical or obstetrical complications except GDM. In race and route of delivery, (%) is the percentage of the total

### Changes in the circulating miRNAs across gestation in NGT and GDM pregnancies

**Fig. 2:**
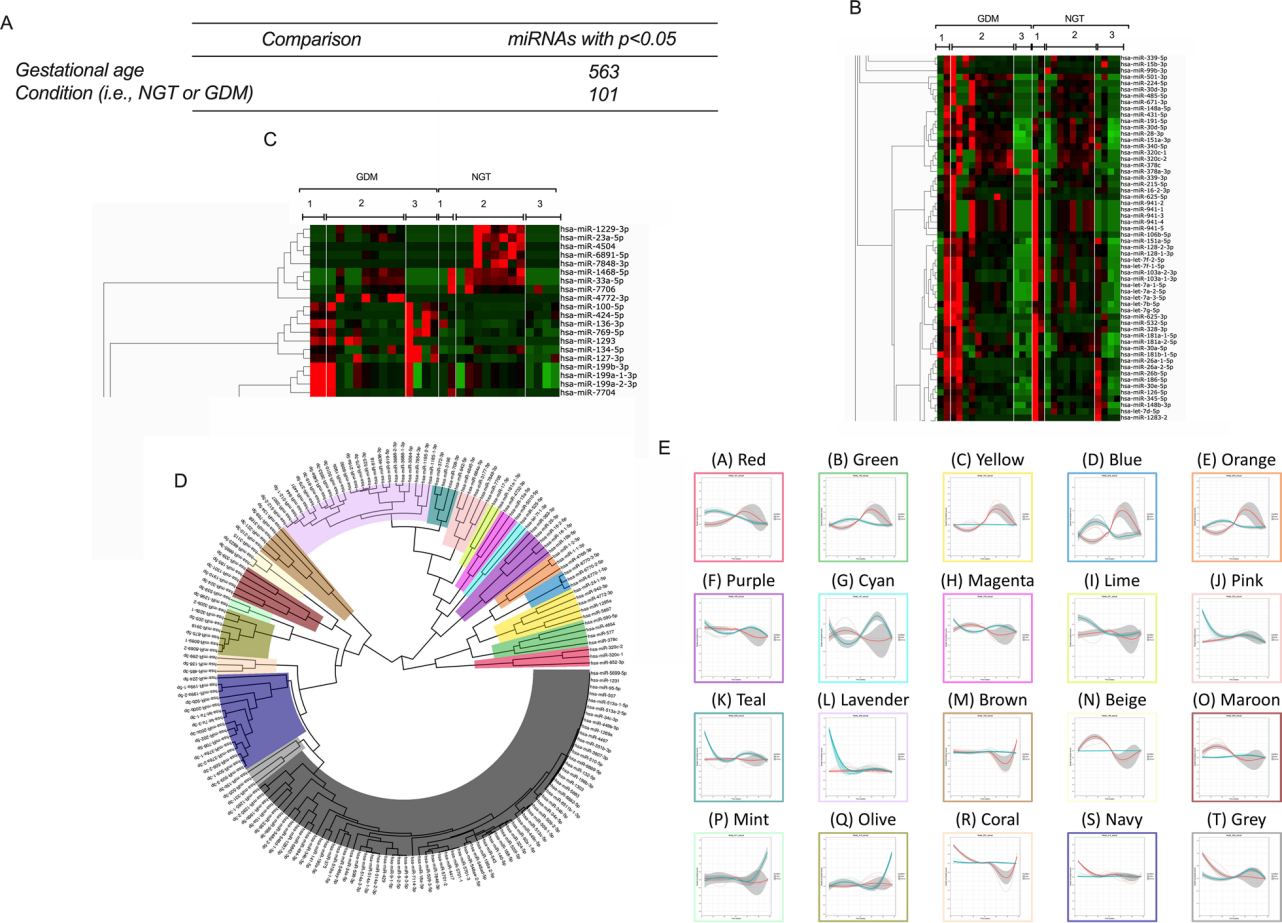
Gestational variation in EV-associated miRNAs across gestation. EVs were isolated from plasma obtained from women with NGT and GDM across gestation in a cross-sectional study design. Analysis was performed on EVs microRNA profiles generated using next-generation sequencing. **A** Number of miRNAs that changed significantly across gestational age, and condition (e.g. NGT or GDM). Heatmap of the top statistically significant miRNAs (adjusted P value of < 0.05) across gestation (**B**), and between NGT and GDM (**C**), where red and green are high and low expression, respectively. **D** Linear mixed modelling of 101 statistically significant miRNAs that change across gestation when comparing normal to GDM pregnancies. miRNA counts were normalized using the DESeq2 package in R, before statistical analysis using the likelihood ratio test. Subsequently, linear mixed modeling was performed on the 101 statistically significant miRNAs (p-value < 0.05) that change across gestation when comparing normal to GDM pregnancies, using the lme4 package in R. The data was scaled between 0 and 1, before hierarchical clustering analysis using Euclidean distance, which is displayed as a circular cladogram (generated using the ggtree package in R). **E** Each color of the circular cladogram represents a different cluster and its trend, as shown in **A**–**T**. Within the panels, the color blue is for normal pregnancies whilst the color red is for GDM pregnancies

Small miRNA sequencing identified a total of 563 sEVs-associated miRNA that were differentially across gestation and 101 EVs miRNAs that different between NGT and GDM. (Fig. 2A). Figure 2A, B presents a hierarchical clustering of the most significant EV miRNAs (p < 0.05) that change across gestation (Fig. 2B), and between NGT and GDM (Fig. 2C). Linear mixed modelling was used to analyze the distribution patterns of EV-associated miRNAs across gestation and in association with GDM (Fig. 2D, E). Hierarchical clustering analysis of the average miRNA expression profiles across gestation identified a number of specific trends. When compared to NGT pregnancies, expression in GDM was: (i) elevated in clusters A, B, C, D and E (number of miRNAs per cluster: A = 3, B = 4, C = 5, D = 3, E = 3 miRNAs) in the third trimester, and clusters N and O (number of miRNAs per cluster, N = 3, O = 5 miRNAs) in the second trimester; (ii) lower in clusters F, G, H, M and R (number of miRNAs per cluster, F = 5, G = 2, H = 3, M = 5, R = 3 miRNAs) in the third trimester (iii) unchanged in clusters I, J, K and L (number of miRNAs per cluster, I = 2, J = 5, K = 4, L = 24 miRNAs) in the second trimester, and clusters P and Q (number of miRNAs per cluster, P = 2, Q = 7 miRNAs) in the third second trimester (iv) decreased in clusters S, and T (number of miRNAs per cluster, S = 15, T = 3) throughout the second trimester. The sEV-associated miRNAs with the greatest expression in each cluster were hsa-let-7i-5p, hsa-miR-10a-5p, hsa-miR-151b, hsa-miR-16–2-3p, has-miR-16-5p, hsa-miR-1910-5p, hsa-miR-423-5p, hsa-miR-92a-3p, hsa-miR-92b-3p, and, and these miRNAs were selected for validation in an independent cohort of patients.

### Quantitative changes in selected EV-associated miRNAs and their association with clinical parameters

**Fig. 3:**
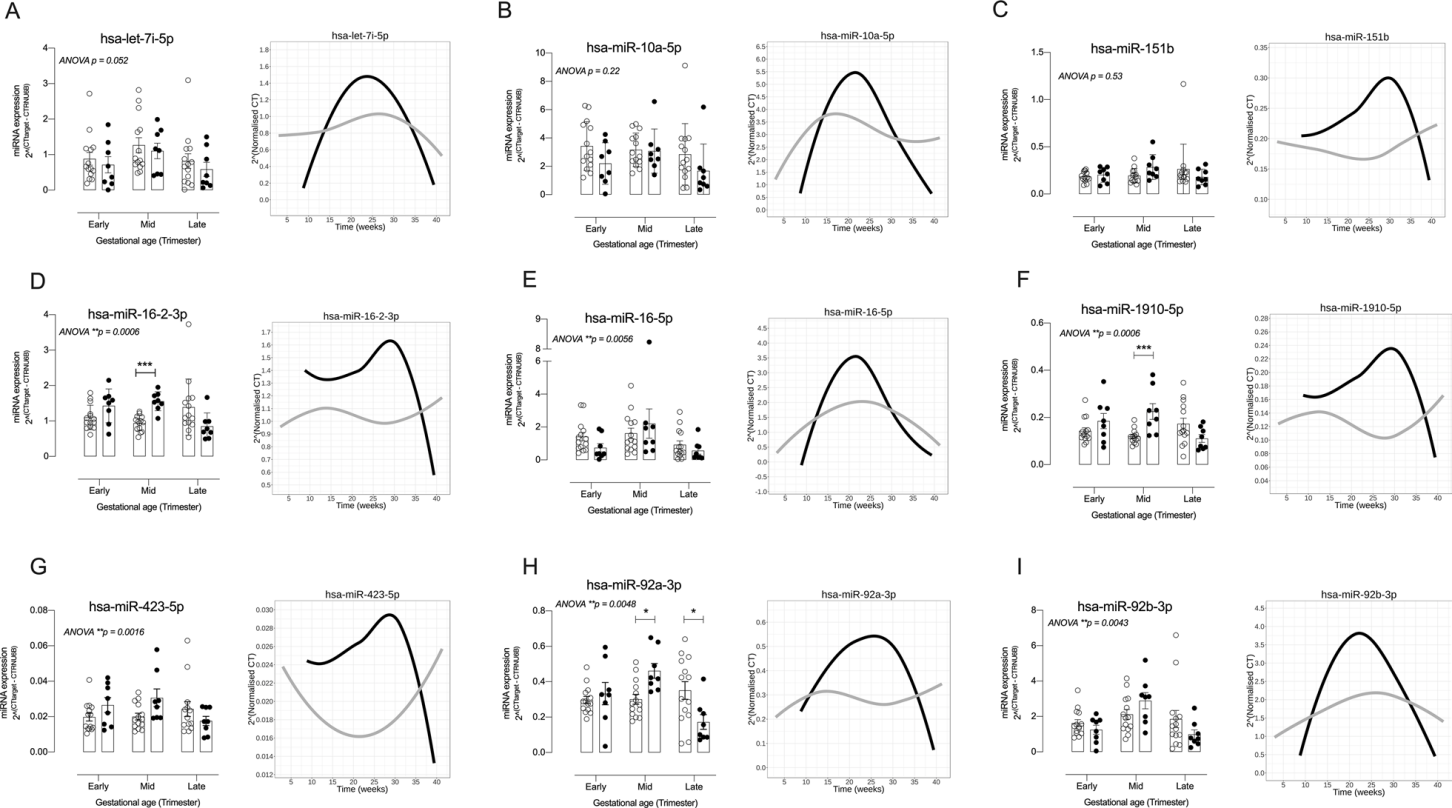
Validation of GDM-associated miRNAs within EVs in a longitudinal independent cohort. The expression of selected miRNAs in EVs was determined in EVs isolated from NGT and GDM pregnancies across gestation. *Left*: data is presented as individual values of the same women at three times during gestation. *Right*: Linear mixed modelling analysis of real-time PCR data for candidate miRNAs for NGT and GDM pregnancies across gestation. Linear mixed modelling (lme4 package in R) was used to analyze the normalized CT values generated from real-time PCR data. The miRNAs were normalized to RNU6B using the ∆CT method, and the normalized values raised to power 2 for easier interpretation of results. Within the panels, the color gray is for normal pregnancies whilst the color black is for GDM pregnancies

Figure 3 presents gestational and GDM-associated quantitative changes in the expression of selected EV-associated miRNA. Longitudinal time-series analysis identified six miRNAs: hsa-miR-16–2-3p (p = 0.0006), hsa-miR-16-5p (p = 0.0056), hsa-miR-1910-5p (p = 0.0006), hsa-miR-423-5p (p = 0.016), hsa-miR-92a-3p (p = 0.048), and hsa-miR-92b-3p (p = 0.0043) that varied significantly across gestation in NGT compared to GDM. Interestingly, pre-gestational BMI was identified as a significant factor in contributing to the variation in expression of miRNA hsa-miR-16–2-3p (p = 0.008), hsa-miR-1910-5p and hsa-miR-92a-3p.

Linear regression analysis was used to identify correlations (independent of GDM status) between: (i) EVs miRNA expression and maternal age, and then (ii) EVs miRNA expression and maternal age at each trimester. Independent of gestational age, no statistically significant effect of maternal age on EVs miRNA expression was identified. When analyzed by gestational age and maternal age, no statistically significant correlations between maternal age and EVs miRNA were identified for early or mid-gestation. In late gestation, statistically significant correlations between maternal age and EVs miRNA were identified for hsa-miR151b (p = 0.02) and hsa-miR423-5p (p = 0.02).

In order to elucidate the potential origin of the miRNAs associated with sEVs, the expression of selected miRNAs was also determined in sEVs isolated from non-pregnant women and compared with the expression in sEVs from NGT and GDM, matched by maternal BMI and age. All the selected miRNAs (hsa-let-7i-5p, hsa-miR-10a-5p, hsa-miR-16–2-3p, has-miR-16-5p, hsa-miR-92a-3p, hsa-miR-92b-3p, hsa-miR-151b, hsa-miR-423-5p, and hsa-miR-1910-5p) are expressed in sEVs from non-pregnant women, suggesting that they are not specific for pregnancy. No statistically-significant differences (p > 0.05, using Kruskal–Wallis test for the non-normally distributed data) in the expression of these miRNAs across non-pregnant women, NGT (average across gestation), and GDM (average across gestation) were identified, with the exception of hsa-miR-10a-5p (*p = 0.027), and hsa-miR-423-5p (*p = 0.031) (Additional file 1: Fig. S3).

### Classification efficiency of maternal plasma EV-associated miRNAs

**Fig. 4:**
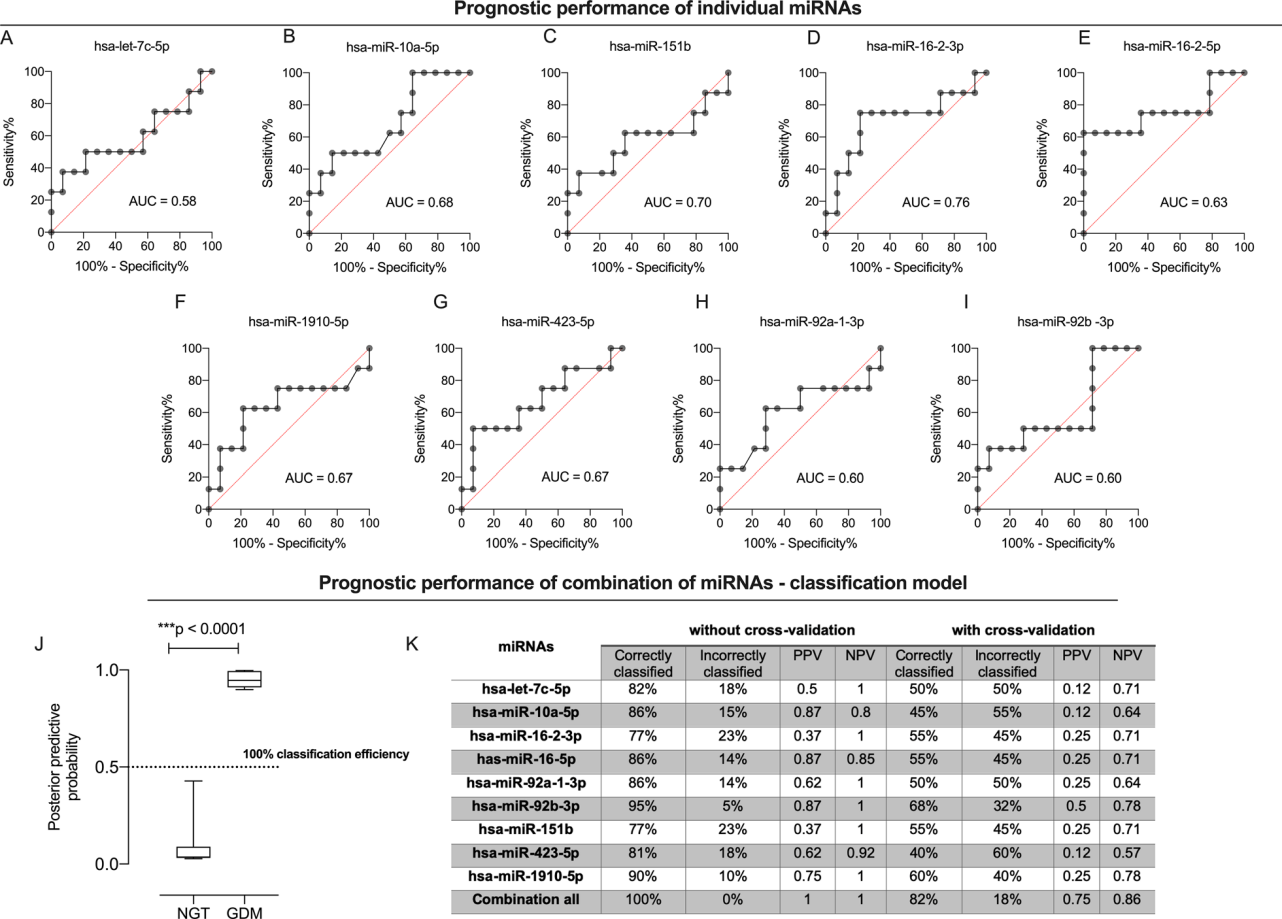
Receiver operating characteristic (ROC) curves illustrating the ability of miRNAS within EVs to distinguish pregnant women at early gestation who will develop GDM later during pregnancy. The predicted likelihood of woman with GDM compared to normal glucose tolerance test at early gestation. **A**–**I** Receiver-Operating Characteristic (ROC) Curves, and the area under the ROC curves (AUC) were calculated using GraphPad Prism (GraphPad Software, Inc., CA, US). **J**–**K** Classification model based on the quantification of EVs miRNAs biomarkers and developed using LogitBoost regression analysis. AUC = 1.0. At a posterior predictive probability threshold of 0.5; sensitivity = 100%, specificity = 100%. **J** The predicted likelihood (posterior predictive probability value) that a woman in first trimester of pregnancy will subsequently develop GDM. **K** ROC and AUC from **J**. **L** Performance of classification models based on the expression of individual miRNAs within sEVs, developed using a machine learning LogitBoost classifier in an external validation dataset. PPV = positive predictive values; NPV = negative predictive values

The classification efficiency (*i.e.* the proportion of cases correctly identified) of measuring the expression of individual miRNAs, and miRNA combinations in circulating sEVs in maternal plasma at early gestation (i.e., < 18 weeks) was assessed by ROC curve analysis (Fig. 4A–I). Next the miRNA classifiers for inclusion in a multivariate index assay were selected based on their individual predictive and degree of redundancy between classifiers [30], as implemented in the WEKA data mining package [31]. An additive logistic regression model (LogitBoost, WEKA), with leave-one-out cross-validation, was developed using the complete data set for the selected classifier. The area under the ROC curve was 1.0, with PPV, and NPV of 1.0 without the cross validation, and for cross validation the area under the ROC curve was 0.80 with PPV, and NPV of 0.75, and 0.85, respectively (Fig. 4J, K). The overall classification accuracy was 100% (i.e., all the women were correctly classified), and 82% (i.e., 8/10 women were correctly classified) without and with cross validation, respectively. The median value (Controls = 0.036; and Cases = 0.94), and interquartile range (Controls: 75th percentile = 0.089, and 25th percentile = 0.031, range = 0.049; Cases: 75th percentile = 0.99, and 25th percentile = 0.91, range = 0.08) for the predicted posterior probability of membership of the disease group for cases and controls, and the performance of classification models based on the expression of individual and combination miRNAs within sEVs is presented in the Fig. 4J. A summary of the ROC curves, positive, and negative predictive values are presented in Fig. 4K. The data support the hypothesis that analysis of miRNAs encapsulated in EVs at early gestation can be used as potential biomarkers to identify women at risk of developing GDM later during gestation.

### Proteomics of skeletal muscle tissue from NGT and GDM

**Fig. 5:**
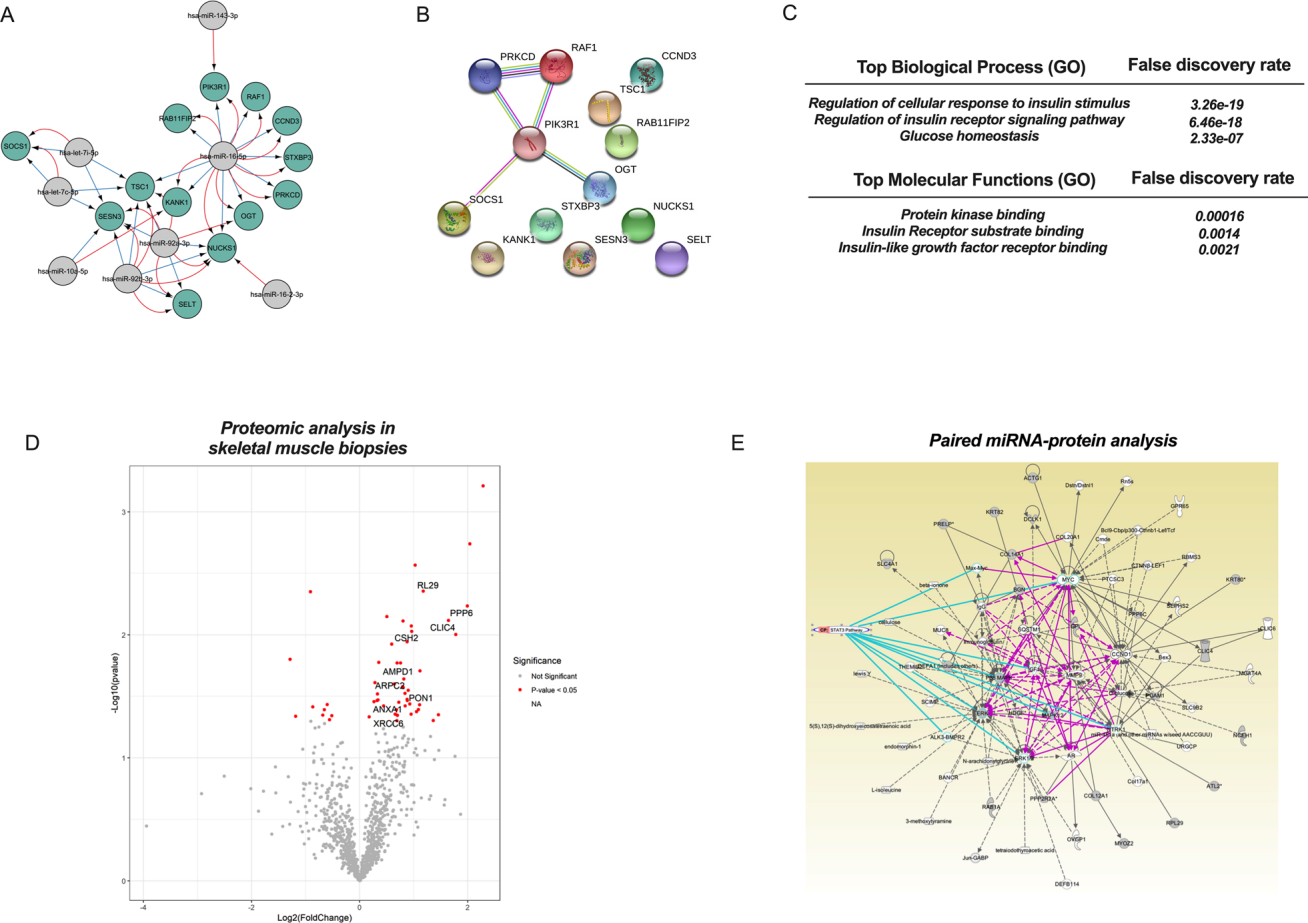
Protein profile in skeletal muscle biopsies and their association with EV miRNAs in GDM. **A**–**C** miRNA gene target and gene ontology analysis for selected miRNAs. Gene ontology analysis for these gene targets showed enrichment for several biological processes. **D** Volcano plot showing differentially expressed proteins in skeletal muscles in GDM compared to NGT. The horizontal axis represents the log2 of fold change and the vertical axis represents the p-value. Each grey dot represents a protein with dots on the right of the zero are proteins upregulated while on the left are downregulated in GDM skeletal muscle. Each red dot represents proteins which are significantly differentially expressed with p value < 0.05. The proteins which are associated with diabetes and targeted by the exosome miRNAs on Pair Analysis are labelled. **E** On integration of EV miRNA and skeletal muscle protein expression profiles, we identified miRNA-targeted networks involved in regulation of skeletal muscle insulin signaling and glucose homeostasis. Each network displays the genes as nodes and the relationships between the nodes as lines. STAT 3 was identified to be a significant pathway targeting by multiple proteins (indicated by blue lines)

Functional bioinformatic analysis identified that the selected miRNAs target genes associated with insulin signalling and glucose homeostasis (Fig. 5A–C). As the skeletal muscle is the principal site for glucose uptake in the body and GDM leads to changes in the insulin signalling and associated molecules in skeletal muscle, we seek to identify potential signalling pathways in skeletal muscles that are associated with the changes in the EV miRNAs across gestation. We performed a quantitative, data-independent acquisition MS analysis in skeletal muscle biopsies obtained from NGT women and women with GDM at the time of term caesarean section. Information Dependent Acquisition (IDA) of mass spectra from off-gel protein and peptide digestion identified 2682 proteins and 55 proteins were differentially abundant in skeletal muscle from GDM patients compared to NGT (p < 0.05) (Fig. 5D). Using an integrative pair miRNA-mRNA analysis (using the proteomic profile) by IPA, we obtained a regulatory network of the signalling pathways associated with the genes of the dysregulated proteins in GDM compared to NGT, that could be targeted by the miRNAs within circulating sEVs across gestation (Fig. 5E). Furthermore, regulatory network mediated by these dysregulated miRNAs predicts diverse effects on the following cellular functions, including glycolysis (p value = 0.00003), gluconeogenesis (p value = 0.00003), and Integrin-Linked Kinase (ILK), and Signal transducer and activator of transcription 3 (STAT3, p value = 0.00003). Interestingly, the integrative paired miRNA (using the miRNA candidates)-mRNA analysis (using the proteomic profile) identified that hsa-miR-92a-3p is associated with dysregulated proteins in skeletal muscle biopsies. Therefore, in the next set of experiments, we focused on the effect of hsa-miR-92a-3p in skeletal muscle cells.

### Effect of miRNA-92a-3p in skeletal muscle cells

**Fig. 6:**
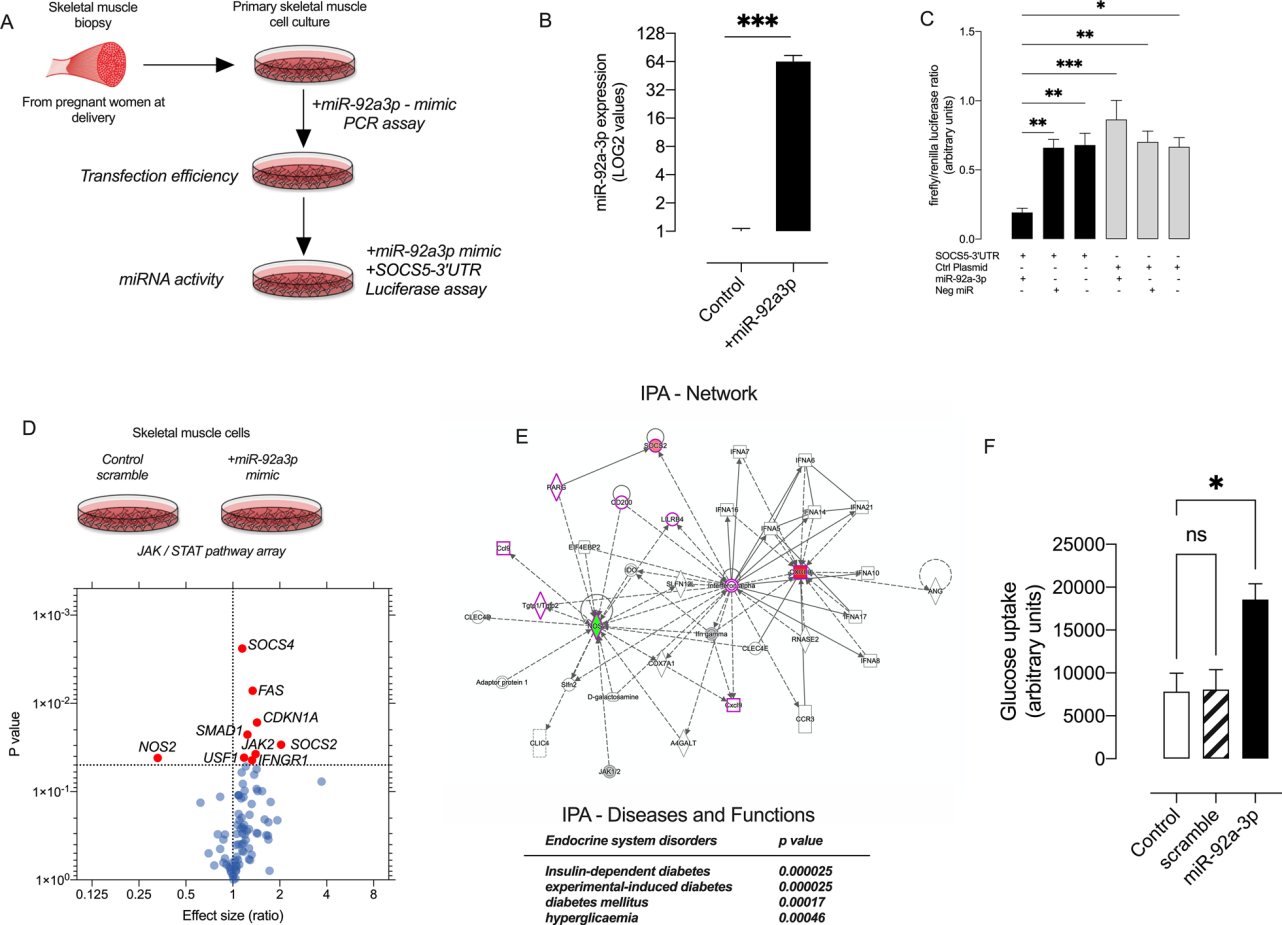
Effect of miR-92a-3p in skeletal muscle cells. **A** Skeletal muscle biopsy samples were obtained from pregnant women at the time of delivery and primary skeletal muscle cell cultures were developed. The skeletal muscle cells were transfected with miR-92a-3p mimic and transfection efficiency and miR-92-3p activity was analysed by SOCS5-3′ UTR luciferase assay **B** miR-92a-3p expression in transfected skeletal muscle cells measured by real-time PCR. miRNA expression is expressed as log2 values of foldchange. **C** Dual luciferase assay of SOCS5 3’UTR plasmid and control plasmid in skeletal muscle cells transfected with miR-92a-3p and negative control miRNA **D** JAK/STAT PCR array after transfection of skeletal muscle cells with miR-92a-3p and control scramble. Volcano plot depicting the genes upregulated and downregulated in cells transfected with miR-92a-3p compared to control miRNA (E) Gene ontology analysis of SOCS2 and NOS2 target proteins Poly(ADP-ribose) glycohydrolase (PARG), C–C motif chemokine 9 (Ccl9), T cell specific GTPase 1 and 2 (Tgtp1&2), Cluster of Differentiation 200 (CD200), Leukocyte immunoglobulin-like receptor subfamily B 4 (LILRB4), Interferon alpha and Chemokine (C–X–C motif) ligand 9 (CXCL9) and pathways associated with diabetes and hyperglycaemia. (F) Insulin-stimulated glucose uptake in skeletal muscle cells transfected with miR-92a-3p and control miRNA and non-transfected cells

The activity of miR-92-3p in primary skeletal muscle cells was analysed using 3′ UTR dual luciferase assay. Dual luciferase assay was performed using plasmid with firefly renilla luciferase system and SOCS5 3′ UTR as well as control plasmid without the SOCS5 3′ UTR (Fig. 6A). Primary skeletal muscle cells were transfected with miR-92a-3p and negative miRNA. Transfection with miR-92a-3p significantly increase the expression of this miRNA (around 64 fold higher) in primary skeletal muscle (Fig. 6B), and reduced SOCS5 expression to < 70% as compared to controls (Fig. 6C). These data confirm that the efficiency and activity of the transfection of miR-92a-3p in primary skeletal muscle cells, and the capacity of this miRNAs to interact with their target genes.

Interestingly, bioinformatic analysis of the protein profile in skeletal muscle and circulating miRNAs within sEVs showed a potential role of JAK/STAT signaling pathway in GDM. Therefore, we evaluated the effect of overexpression of miR-92a-3p in skeletal muscles on the expression of 84 genes associated with JAK/STAT signaling. We identified a total of 9 genes (Suppressor of cytokine signaling 2 (SOCS2), Mothers against decapentaplegic homolog 1 (SMAD1), Upstream stimulatory factor 1 (USF1), Cyclin-dependent kinase inhibitor 1 (CDKN1A), TNF receptor superfamily member 6 (FAS), Suppressor of cytokine signaling 4 (SOCS4), Nitric oxide synthase, inducible (NOS2), and Janus kinase 2 (JAK2)) significantly differentially expressed in cells transfected with miR-92a-3p compared to negative miRNA (p < 0.05) (Fig. 6D). miR-92a-3p increased the expression of SOCS4 (1.2-fold), FAS (1.4-fold), CDKN1A (1.5-fold), SMAD1 (1.3-fold), SOCS2 (2.2-fold), JAK2 (1.4-fold), USF1 (1.2-fold), and IFNGR1 (1.4-fold). Interestingly, miR-92a-3p decreased (> 65%) the expression of NOS2, negative 3.08-fold. Bioinformatics analysis using IPA showed that the genes that were differentially expressed in the PCR array were targeting pathways associated with insulin-dependent diabetes, experimental-induced diabetes, diabetes mellitus and hyperglycemia (Fig. 6E). Next, we evaluate whether miR-92a-3p can modify the glucose uptake in response to insulin in skeletal muscle cells. Over expression of miR-92a-3p increased around 2.3fold higher the glucose uptake in the presence of insulin in primary skeletal muscle cells compared with controls (i.e., cells transfected with negative control miRNA or non-transfected cells) (Fig. 6F).

## Discussion

The principal findings of this study are: (i) we developed a classification model that combines the measurement of nine miRNAs in sEVs, that displays appropriate classification performance based on cross-sectional samples (*i.e.* case–control comparisons). Using a leave-one-out cross-validation, both models can correctly classify at least 82% of the samples, demonstrating the robustness of the biomarker panel, and that both models tested deliver similar results with only minimal loss of performance, (ii) bioinformatic analysis revealed that the EV-associated miRNAs identified as changing with gestational age and GDM belonged to the following biological processes: glycolysis (p value = 0.00003), gluconeogenesis (p value = 0.00003), and Integrin-Linked Kinase (ILK), and Signal transducer and activator of transcription 3 (STAT3, p value = 0.00003), and iii) overexpression of hsa-miR-92a-3p in primary skeletal muscle cells (pSKM) regulates pathways associated with insulin-dependent diabetes, experimental-induced diabetes, diabetes mellitus and hyperglycemia, and enhances insulin-dependent glucose uptake in pSKM.

GDM is a serious public health issue affecting 9–15% of all pregnancies worldwide. This complication of pregnancy not only causes acute adverse pregnancy outcomes for mother and infant, but also increases the lifetime risk of the infant developing metabolic syndromes (including obesity and type II diabetes), and of type II diabetes in the mother. Moreover, a female born from a GDM pregnancy has a higher chance of developing GDM during her pregnancy, thus, creating a recurring disease cycle. Identification of women who are at a higher risk of developing GDM during the first trimester of pregnancy, provides the opportunity for early life style intervention and better treatment options. This will reduce the incidence and/or severity of this complication and hence improved pregnancy outcomes. Also, the prevention of recurring intergenerational transmission of metabolic syndrome can help in tackling the global burden of diabetes and associated comorbidities.

Recently, EVs have been identified as an important mediator of cell-to-cell communication under normal and pathological conditions, including GDM [5, 7, 32, 33]. sEVs like exosomes are enriched in miRNAs, and in this study, we comprehensively analyzed the sEV miRNA profile across gestation in NGT and GDM pregnancies. Using next generation sequencing, we identified dramatic changes in a set of miRNAs encapsulated in the circulating sEVs in GDM compared to NGT across gestation. We measured the expression of nine candidate miRNAs using qRT-PCR in an independent cohort of patients as validation phase and identified significant differences in GDM compared to NGT pregnancies across gestation. Interestingly, bioinformatic analysis of these miRNAs showed that they might be involved in the regulation of genes associated with insulin signaling and glucose homeostasis. As difference in the miRNA content within EVs at early pregnancy were observed, we developed a classification model using the expression of the miRNAs hsa-miR-let7i-5p, hsa-miR-10a-5p, hsa-miR-151b, hsa-miR-16–2-3p, hsa-miR-16-5p, hsa-miR-1910-5p, hsa-miR-423-5p, hsa-miR-92a-3p, and hsa-miR-92b-3p at early gestation, which can predict the risk of developing GDM later in pregnancy, with a classification efficiency of 100% and 82% for without and with cross validation, respectively (using machine learning and LogitBoost algorithm).

In this study, we profiled the expression of over 2000 miRNAs and identified that the miRNAs, hsa-miR-16-2-3p, hsa-miR-16-5p, hsa-miR-1910-5p, hsa-miR-423-5p, hsa-miR-92a-3p, and hsa-miR-92b-3p are differentially expressed in GDM compared to NGT pregnancies. The expression of some of these miRNAs, such as hsa-miR-423-5p and hsa-miR-16-2-3p is reported to be altered in type 2 diabetes [34, 35]. Karolina et al. reported that hsa-miR-92a-3p regulates insulin biosynthesis and the stability of insulin mRNA, leading to decreased insulin synthesis [36]. In our study, hsa-miR-92a-3p is one of the highly expressed miRNAs in the circulating sEVs, and is upregulated in the second and third trimester, in NGT and GDM pregnancies, respectively. Insulin secretion rates and serum insulin concentrations are lower in GDM in late pregnancy compared to their NGT controls [37] and this is confirmed for hsa-miR-92a-3p expression in our study. Furthermore, hsa-miR-92a-3p is upregulated in placenta of macrosomic newborns [38], which is a common complication of GDM. The authors, however, do not mention if there is a relationship between this miRNA and GDM status.

Currently, there is no reliable early detection test available to identify pregnant women who are at the risk of developing GDM. The most clinically relevant solution to this problem is to develop, validate and implement an early pregnancy, blood-based screening test to identify women at high risk of developing GDM 39–44. Thus, in this study, we developed a classification model that combines the measurement of nine miRNAs in sEVs, that displays appropriate classification performance based on cross-sectional samples (i.e., case-control comparisons, Fig. 3). Moreover, using a leave-one-out-cross-validation, both models can correctly classify at least 85% of the samples, demonstrating the robustness of the biomarker panel, and that both models tested deliver similar results with only minimal loss of performance.

In our study, the expression of hsa-miR-92a-3p, hsa-miR-16-2-3p and hsa-miR-1910-5p within circulating sEVs were upregulated in GDM, and their expression changed with increasing maternal BMI. Obesity is the strongest risk factor for GDM and women who are overweight and obese have decreased insulin sensitivity compared to lean women [45]. There are previous reports on the association of these miRNAs with obesity and metabolic disorders, and the strongest association has been identified between circulating EV concentrations of hsa-miR-92a-3p and obesity. Specifically, the concentration of hsa-miR-92a-3p in circulating small EV is inversely correlated with the metabolic activity of brown adipose tissue [46], which in turn indicates impaired metabolic status and insulin sensitivity [47]. The association between the other miRNAs within small EV and maternal BMI has not been previously described. Interestingly, hsa-miR-1910-5p has been identified to be involved in the regulation of obesity by modulating the conversion of white adipose tissue to brown adipose tissue, by targeting genes such as fibronectin type III domain containing (FNDC), peroxisome proliferator-activated receptor (PPAR), and PR domain containing (PRDM) [48].

Skeletal muscle is the main site of insulin-mediated glucose uptake and this response to insulin is reduced in insulin resistant states [49]. We performed quantitative proteomics of skeletal muscle from NGT and GDM women using MS-SWATH. We identified 55 proteins to be differentially expressed in GDM indicating novel alterations of skeletal muscle proteome in GDM. Among these, Paraoxonase-1 (PON1), AMP deaminase-1 (AMPD1) and Annexin 1 (ANXA1) are involved in diabetes or GDM. PON1 is an HDL- associated anti-oxidant and reduced PON1 activity has been associated with diabetes and its cardiovascular complications [50]. In high fat diet fed mice, PON1 increases the GLUT4 expression in skeletal muscles by increasing the tyrosine phosphorylation of IRS-1 molecule [51]. In contrast, our study identified a higher expression of this enzyme in skeletal muscle in GDM. Muscle specific AMPD1 regulate multiple aspects of insulin sensitivity and insulin action in skeletal muscle [52] and had an enhanced expression in skeletal muscle in GDM as per our study which corroborates with the current findings. We found an increased expression of ANXA1 in skeletal muscle in GDM and increased levels of ANXA1 in serum has been reported in obesity-related type 2 diabetes mellitus [53]. The exosomal miRNAs upregulated in GDM were identified to target some of these differentially expressed proteins and the most important are the 60S Ribosomal Protein L29 (RL29), Serine/Threonine Protein Phosphatase 6 (PPP6), Chloride Intracellular Channel Protein 4 (CLIC4) and Actin Related Protein Complex 2 (ARPC2) by miRNAs hsa-miR-1910-5p, hsa-miR-16-5p, hsa-miR-92a-3p and hsa-miR-92-3p respectively. The skeletal muscle proteins targeted by the sEVs miRNAs, target regulatory pathways associated with glucose metabolism and insulin signalling. The most important of these is the STAT3 pathway, involved in the cytokine and nutrient-mediated insulin resistance in skeletal muscles [54]. Interestingly, sEVs miRNA-mediated downregulation of STAT3 and impaired insulin signalling in skeletal muscles has been reported in type 2 diabetes [55]. Moreover STAT3 acts as link between obesity and diabetes by mediating lipid-induced insulin resistance [56]. In this sense, the differential miRNA profile in exosomes and their target proteins in the skeletal muscles may contribute to the pathophysiology of GDM.

Our findings from the PCR array shows that miR-92a-3p induces SOCS2 and suppress NOS2 expression in skeletal muscle cells. SOCS proteins can downregulate the cytokine or tyrosine kinase receptor signalling by targeting signaling proteins such as JAK and IRS family members [57]. Increase levels of pro-inflammatory cytokines such as IL-1β, IL-6, TNF-α and growth hormone is an important characteristic of peripheral insulin resistance [58]. It has been reported that SOCS-1 and SOCS3 are associated with insulin resistance in various cells through degradation of IRS-1 and IRS-2 [59, 60]. Even though, SOCS-2 is reported to be interacting with the insulin receptor on a yeast two hybrid system [61], a direct link of SOCS2 to insulin sensitivity is not clearly known. SOCS2 protein can interact with Insulin-like Growth Factor-1 (IGF-1) receptor and regulate its signalling [62, 63] and IGF-1 acts in concert with insulin in the regulation of glucose uptake and metabolism. Also, SOCS2 can inhibit the growth hormone signalling in skeletal muscle and reduce muscle mass and SOCS2 knockout is associated with gigantism in mice, indicating its effect on growth hormone/IGF-1 axis [62]. But the overall role of SOCS2 in regulation of insulin sensitivity remains unclear. As per our study, miR-92a-3p decreases the expression of NOS2 in skeletal muscle. NOS2 is part of the innate immune response of cell and regulate the interferon gamma signalling in cells. When IFNγ attaches to its receptor, it activates JAK and then STAT proteins which translocates to nucleus and transcribe the interferon response transcription factor that increase the transcription of NOS. NOS then translocates to cytoplasm and produce nitric oxide that fight against bacteria and virus [64]. The skeletal muscle NOS activity is reported to be impaired in type 2 diabetes indicating its role in muscle glucose transport [65]. Insulin resistant states such as type 2 diabetes is associated with impaired vasodilation in vascular tissue and this is attributed to impaired NO generation by NOS [66]. Overall, these finding indicate that miR-92a-3p has a mechanistic role in regulating the insulin sensitivity in skeletal muscle. Interestingly, from our glucose uptake assay, the skeletal muscle showed an increase in glucose uptake in the presence of miR-92a-3p compared to negative miRNA. In the context of insulin resistance, the effect of miR-92a in glucose uptake in insulin target cells such as liver, skeletal muscle or adipocytes has not been reported yet. However, miR-92a is reported to help beta islet cell repair and restoration [67, 68]. Hence, the increased level of miR-92a-3p in circulating EVs in GDM indicates the miR-92a-3p might be involved in a protective mechanism to prevent hyperglycaemia by increasing the glucose uptake in skeletal muscle cells.

## Strengths and limitations of this study

The study’s major strength is the characterisation of miRNAs within small extracellular vesicles in maternal plasma in normal and GDM pregnancies, in two independent cohorts of patients. The validation phase of this study was performed in longitudinal samples of patients who had a normal pregnancy outcome, or GDM defined by clinical outcomes. We simultaneously examined a large number of miRNAs (> 2800) using small RNA sequencing, a protocol that has been rigorously validated in our laboratory. Another strength of our study is the ability to enrich and characterise extracellular vesicles.

One limitation of this study is the presence of potential confounding factors when interpreting the data presented herein, including small sample size, the unavailability of glycemic status of women pre-pregnancy or in early pregnancy, validation cohort were not age-matched, and different criteria used for the clinical diagnosis of GDM. Another limitation of this study is the lack of comprehensive data about the mechanisms associated with the effect of a selected groups of miRNAs, including hsa-miR-92a-3p encapsulated in small EVs, in the physiopathology of GDM. Therefore, further functional studies are required to determine the role and signalling pathways associated with EV-associated miRNAs in GDM.

## Conclusions

The data obtained in this study are consistent with the hypothesis that maternal plasma sEV-associated miRNAs vary with gestational age and are differentially expressed in association with GDM. During early pregnancy (*i.e.* less than 18 weeks of pregnancy), differently-expressed, EV-associated miRNAs may be of clinical utility in identifying presymptomatic women who will subsequently develop GDM later in gestation. Currently, there is no reliable early detection test available to identify pregnant women who are at risk of developing GDM. The most clinically relevant solution to this problem is to develop, validate, and implement an early pregnancy, blood-based screening test to identify women at high risk of developing GDM. Thus, the data obtained to date warrant the conduct of a larger, prospective biomarker trial to robustly assess the prognostic performance of the EV-miRNA-based multivariate index assay. EV-associated miRNAs were further shown to regulate protein expression in skeletal muscles. Via this mechanism, EV miRNAs may effect insulin resistance and maternal metabolism associated with GDM. In particular, in GDM EV-associated miR-92a-3p altered JAK/STAT signalling and increased insulin sensitivity in skeletal muscle cells (as measure by in vitro insulin-stimulated glucose uptake). We hypothesis that miR-92a-39 containing EV may be released from the placenta as an adaptive or rescue response, by the fetus, to maternal hyperglycaemia.

## Supplementary Information

**Additional file 1.** Additional figures and tables.

## Data Availability

All data generated or analysed during this study are included in this published article [and its additional information files].

## Abbreviations

NGT: Normal glucose tolerant
GDM: Gestational diabetes
PPAR: Peroxisome proliferator receptor;
PHT: Primary human trophoblasts
OGTT: Oral Glucose Tolerance Test
ADIPS: Australian Diabetes in Pregnancy Society
WHO: World Health Organization
TSG: Tumor susceptibility gene
WHO: World Health Organization
NDDG: National Diabetes Data Group
NTA: Nanoparticle tracking analysis
GEO: Gene expression omnibus
BMI: Body Mass Index
ILK: Integrin-linked kinase
STAT3: Signal transducer and activator of transcription 3
qRT-PCR: Quantitative real time PCR
FNDC: Fibronectin type III domain containing
PRDM: PR domain containing
MS: Mass Spectrometry
SWATH: Sequential Windowed Acquisition of All Theoretical Mass Spectra
PON1: Paraoxonase-1
AMPD1: AMP deaminase-1
ANXA1: Annexin 1
HDL: High Density Lipid
GLUT4: Glucose Transporter 4
IRS-1: Insulin Substrate Receptor-1
RL29: 60S Ribosomal Protein L29
PPP6: Serine/threonine protein phosphatase 6
CLIC4: Chloride intracellular channel protein 4
ARPC2: Actin related protein complex 2
SOCS: Suppressor of cytokine signaling 2
IFNGR1: Interferon gamma receptor 1
JAK2: Janus kinase 2
USF1: Upstream stimulatory factor 1
CDKN1A: Cyclin dependent kinase inhibitor 1A
SMAD1: SMAD Family Member 1
NOS: Nitric oxide synthase
NO: Nitric oxide
