# Supplementary material for: Extracellular vesicle-associated miRNAs are an adaptive response to gestational diabetes mellitus
Source: J Transl Med. 2021 Aug 20;19:360. doi: 10.1186/s12967-021-02999-9 (PMC8377872; doi:10.1186/s12967-021-02999-9)
Supplement: Supplementary file 1 — Additional file 1. Additional figures and tables. [file 12967_2021_2999_MOESM1_ESM.docx]

***
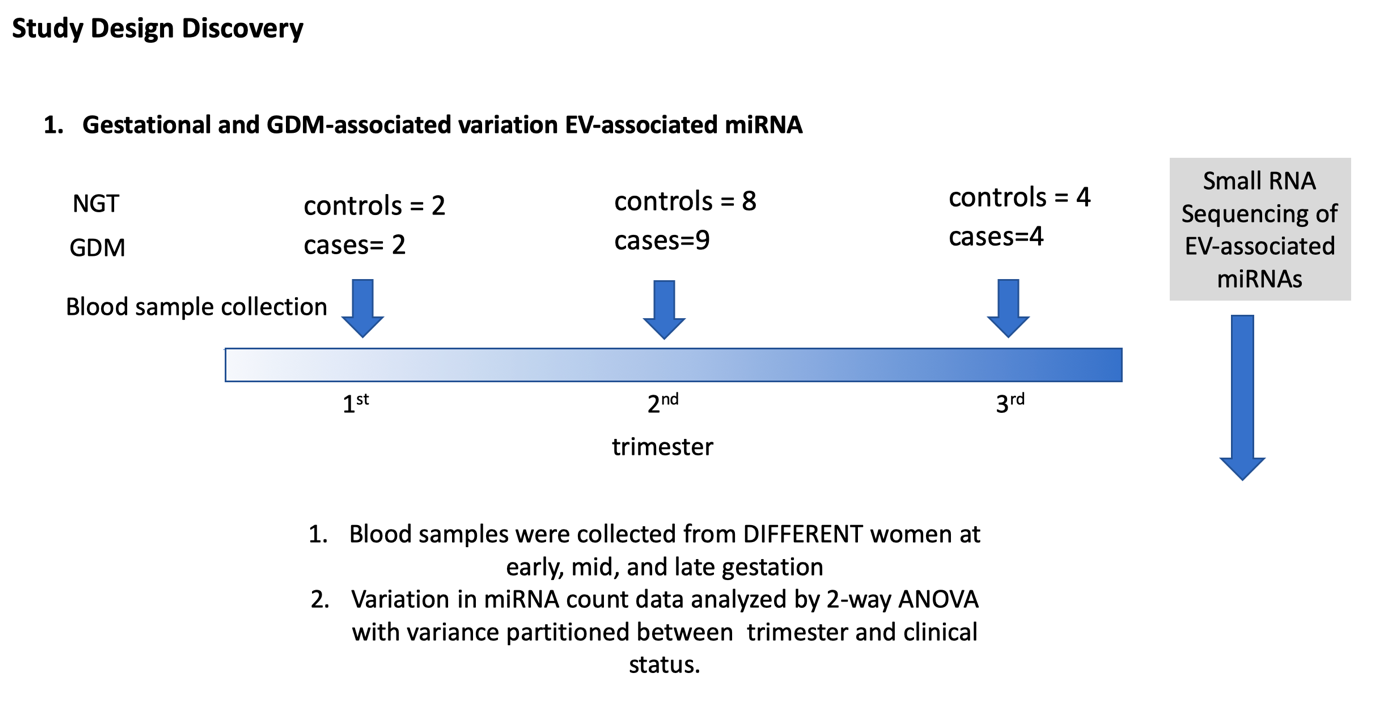
***

**Figure S1. Experimental design of the discovery cohort.**T*o characterize gestational and GDM-associated variations in EV miRNA profiles, a retrospective, case-control study design was used. EVs were isolated from maternal plasma obtained at early (cases=2; controls=2), mid (cases=9; controls=8), and late (cases=4; controls=4) gestation and small RNA sequencing was used to identify EV-associated miRNAs transcripts. Variation in miRNA expression (i.e. count data were analyzed by ANOVA with variance partitioned between trimester and clinical status).*

*
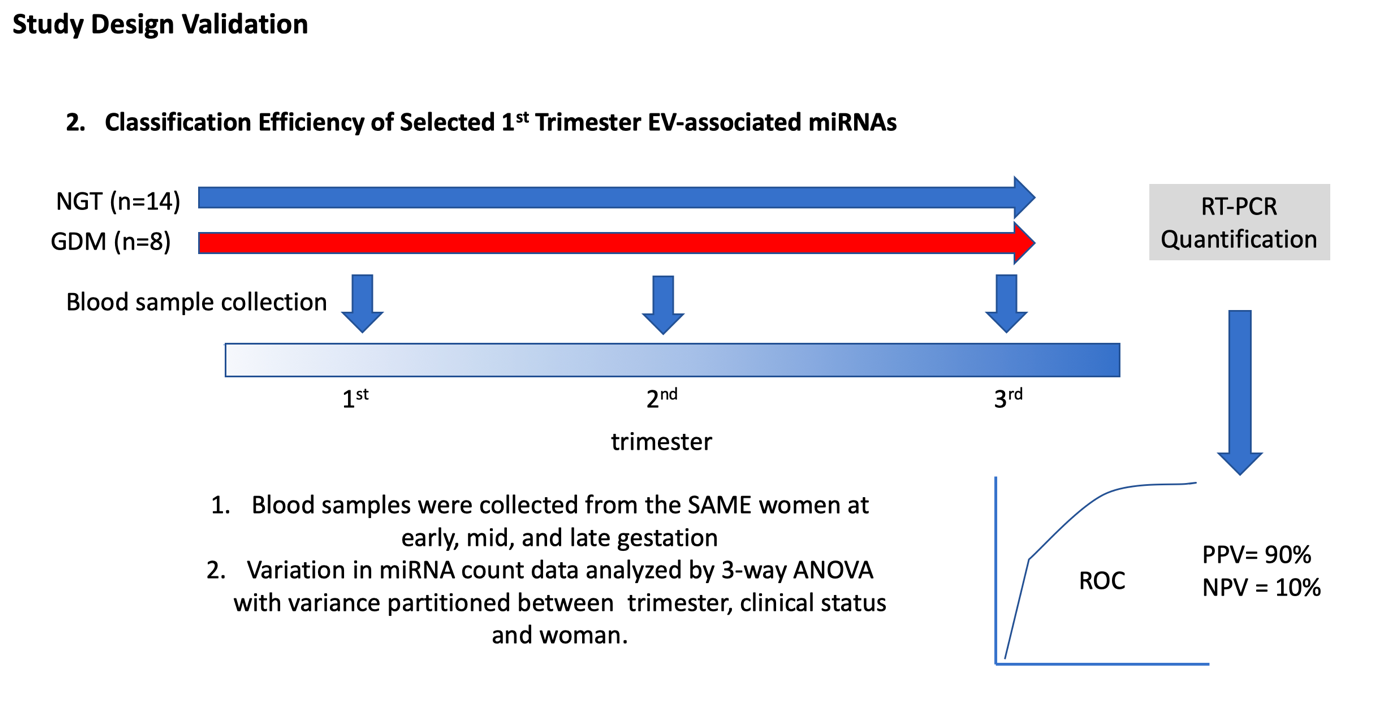
*

**Figure S2. Experimental design of the validation cohort.** To validate data obtained in the cross-sectional study and to determine the efficiency of miRNAs present in EVs to correctly classify samples, a retrospective, longitudinal, case-control study design was used, in which selected miRNAs were quantified by real time PCR (cases = 8, control = 14, sampled at three times during pregnancy) and their individual and combined.

**Supplementary Table 1**

Power Calculation

| Comparison | GDM FT vs ST | GDM FT vs TT | GDM ST vs TT | NGT FT vs ST | NGT FT vs TT | NGT ST vs TT |
| --- | --- | --- | --- | --- | --- | --- |
| hsa-miR-1910-5p | 0.48 | - | - | 0.52 | - | - |
| hsa-miR-423-5p | - | 0.96 | 0.94 | - | 0.5 | 0.3 |
| hsa-miR-92b-3p | 1 | 1 | 0.99 | 0.81 | 0.8 | 0.01 |
| hsa-miR-151b | 1 | 0.54 | 0.99 | 0.96 | 0.1 | 0.99 |
| hsa-let-7i-5p | 0.9 | 0.96 | 0.6 | 0.36 | 0.92 | 0.4 |
| hsa-miR-10a-5p | 1 | 1 | 0.99 | 0.96 | 0.89 | 0.54 |
| hsa-miR-143-3p | 1 | 0.99 | 0.1 | 1 | 1 | 0.97 |
| hsa-miR-16-2-3p | 0.99 | 1 | 0.99 | 0.99 | 0.99 | 0.25 |

Here we calculate the achieved power for some miRNAs using the mean and standard deviation from the miRNA RNAseq data. We selected the miRNAs that were used in the longitudinal study. Below is the estimated statistical power using T-test: significance level = 0.05 and a sample size of 29 (15 GDM & 14 NGT). GDM = Gestational Diabetes Mellitus; NGT = Normal Glucose Tolerance Test; FT = First Trimester (i.e., early gestation); ST = Second Trimester (i.e., mid gestation); TT = Third Trimester (i.e., late gestation).

**Supplementary Table 2**

Clinical characteristics of patients and newborns in the discovery cohort

|  | NGT  (n=15) | GDM  (n=14) |
| --- | --- | --- |
| Maternal baseline characteristics | | |
| Age (years) | 30.17 ± 11.6 (25-46) | 31.5 ± 4.2 (26-34) |
| Weight (kg) | 77.5 ± 36.2 (63- 103.5) | 78.25 ± 24.2 (62-90) |
| Height (cm) | 165.6 ± 14.6 (155-173) | 162.2 ± 9.6 (155-171) |
| BMI at delivery (kg/m^2^) | 29.81 ± 8.2 (26-35) | 29.83 ± 6.0 (26-35) |
| Screening and diagnostic results | | |
| Fasting OGTT (mmol/ L) | 4.6 ± 0.7 (3.8-4.9) | 5.21 ± 0.7 (4.6- 5.7) |
| 1hr OGTT (mmol/ L) | 7.2 ± 1.5 (6.0-8.7) | 9.31 ± 3.9 (5.0- 12.4) |
| 2hr OGTT (mmol/ L) | 5.6 ± 1.9 (3.6-7.0) | 8.28 ± 2.1 (5.4-9.3)* |
| Delivery data | | |
| Gestational age at delivery (weeks) | 39.97 ± 1.74 (39-41.6) | 39.35 ± 0.8 (38.5-40) |
| Fetal weight (g) | 3783 ± 376 (3450-4060) | 3514 ±432 (3160-3940) |
| Fetal sex (male/female) | 10/8 | 9/9 |

Data are presented as mean ± SD (range). All pregnancies were normotensive, and without intrauterine infection or any other medical or obstetrical complications except GDM. **p*<0.05 versus Normal. OGTT: oral glucose tolerance test; BMI: body mass index. NGT: women with normal tolerance test, and GDM: women with gestational diabetes mellitus.

**Supplementary Table** 3

Size distribution of the exosomes during pregnancy.

| **Discovery cohort** | | |
| --- | --- | --- |
| **Gestational age** | **NGT (n=14)** | **GDM (n=15)** |
| Early (10-14 weeks) | 105 ± 31 nm | 102 ± 55 nm |
| Mid (22-28 weeks) | 103 ± 38 nm | 108 ± 41 nm |
| Late (32-26 weeks) | 111 ± 41 nm | 107 ± 43 nm |
| **Validation cohort** | | |
| **Gestational age** | **NGT (n=14)** | **GDM (n=8)** |
| Early (10-14 weeks) | 102 ± 41 nm | 108 ± 35 nm |
| Mid (22-28 weeks) | 111 ± 28 nm | 118 ± 31 nm |
| Late (32-26 weeks) | 115 ± 51 nm | 117 ± 33 nm |

The size distribution of exosome preparations was analysed using a NanoSight NS500 system (NanoSight, Amesbury, UK) according to the manufacturer’s instructions (see methods). Data are presented as mean ± SD. All preparations were analysed in duplicate and 5 videos were recorded for normal and GDM pregnancy.

**Figure S3. Expression of miRNAs within Extracellular vesicles from non-pregnant women.** Kruskal-Wallis test was used for the non-normally distributed data per miRNA.
